# SGLT2 Inhibition in Heart Failure: Clues to Cardiac Effects?

**DOI:** 10.1097/CRD.0000000000000637

**Published:** 2024-01-08

**Authors:** Patrick Savage, Lana Dixon, David Grieve, Chris Watson

**Affiliations:** * From the Royal Victoria Hospital Cardiology Department; † Wellcome-Wolfson Institute for Experimental Medicine, Queen’s University, Belfast, Northern Ireland.

**Keywords:** SGLT2 inhibitors, heart failure, mechanisms, cardioprotective

## Abstract

Following the publication of several landmark clinical trials such as dapagliflozin in patients with heart failure and reduced ejection fraction, dapagliflozin evaluation to improve the lives of patients with preserved ejection fraction heart failure, and empagliflozin outcome trial in patients with chronic heart failure with preserved ejection fraction, sodium-glucose cotransport 2 inhibitors have been rapidly incorporated as a guideline-directed therapy in the treatment of heart failure. Moreover, their benefits appear to extend across the spectrum of left ventricular dysfunction which in some respects, can be seen as the holy grail of heart failure pharmacotherapy. Despite its plethora of proven cardioprotective benefits, the mechanisms by which it exerts these effects remain poorly understood, however, it is clear that these extend beyond that of promotion of glycosuria and natriuresis. Several hypotheses have emerged over the years including modification of cardiovascular risk profile via weight reduction, improved glucose homeostasis, blood pressure control, and natriuretic effect; however, these mechanisms do not fully explain the potent effects of the drug demonstrated in large-scale randomized trials. Other mechanisms may be at play, specifically the down-regulation of inflammatory pathways, improved myocardial sodium homeostasis, modulation of profibrotic pathways, and activation of nutrient deprivation signaling pathways promoting autophagic flux. This review seeks to summarize the cardioprotective benefits demonstrated in major clinical trials and provide a succinct review of the current theories of mechanisms of action, based on the most recent evidence derived from both clinical and laboratory data.

## Discovery of Sodium-Glucose Cotransport 2 Inhibitors

Sodium-glucose cotransport 2 (SGLT2) inhibitors have been rapidly incorporated as a guideline-directed therapy in numerous fields across the spectrum of clinical medicine. This widespread adoption is built upon a foundation of expanding evidence from randomized controlled trial data, a trend that has been most keenly noticed in cardiovascular (CV) medicine.^1^

Interest in this drug class, termed gliflozins, extends back to the early 1800s when the compound phlorizin was first isolated from the roots of apple trees by De Koninck and Stas and found in early canine models to produce glycosuria. Its use in humans was limited by poor oral bioavailability and marked gastrointestinal side effects owing to the rapid gastrointestinal hydrolysis of the compound in the gut.^2^

Renewed interest by De Fronzo et al. in the 1980s found that administration of phlorizin to insulin-resistant diabetic rats normalized insulin sensitivity and glucose homeostasis; however, it wasn’t until the advent of receptor cloning and the subsequent identification of specific renal and intestinal SGLT2 receptors in the early 1990s, that real translational discovery could take place.^3^ The first iterations of these drugs; namely canagliflozin, empagliflozin, and dapagliflozin, were based on aryl-glucosides compounds which, unlike the O-glucoside phlorizin compound, would not be hydrolyzed by the intestinal brush border lactase thus circumventing the initial difficulty of rapid gastrointestinal hydrolysis. Although based on the same meta-C-glycosylated diarylmethane pharmacophore, these compounds would vary slightly in their respective affinity for SGLT2/SGLT1 receptors.^4^

## Cardiovascular Outcome Trials

SGLT2 inhibitors were first developed for the treatment of type 2 diabetes mellitus (T2DM). Controversy surrounding the previous thiazolidinedione class antidiabetic medication, rosiglitazone, which in postmarketing surveillance was found to increase rates of CV death, dictated that all new antidiabetic medications were mandated to undergo CV outcome trials prior to FDA approval. This led to the *EMPA-REG OUTCOME* (empagliflozin cardiovascular outcome event trial in type 2 diabetes mellitus) trial which was a multicenter randomized 1:1:1 placebo-controlled trial (n = 7028, mean age 63.1 years, 3.1 years follow-up) comparing CV outcomes in patients with diabetes and established CV disease treated with empagliflozin. The primary composite outcome of CV death, nonfatal myocardial infarction (MI), or stroke was significantly reduced in the treatment group [10.5% vs 12.1%; Hazard ratio (HR), 0.86; 95% Confidence interval (CI), 0.74–0.99; *P* < 0.001). Furthermore, reductions in all-cause mortality (3.8% vs 5.1%, *P* < 0.01) and heart failure (HF) hospitalization (2.7% vs 4.1%, *P* = 0.001) were also noted, findings that were entirely unexpected.^5^ This serendipitous discovery led to several landmark confirmatory CV outcomes trials including *CANVAS* (canagliflozin and cardiovascular and renal events in type 2 diabetes) and *DECLARE-TIMI* (dapagliflozin effect on cardiovascular events: a multicenter, randomized, double-blind, placebo-controlled trial to evaluate the effect of dapagliflozin 10 mg once daily on the incidence of cardiovascular death, myocardial infarction, or ischemic stroke in patients with type 2 diabetes), which demonstrated similar findings, leading to the assessment that these favorable outcomes were attributable to a class effect of SGLT2 receptor inhibition.^6,7^

Interestingly SGLT2i was shown to reduce the risk of HF hospitalization by 23% [HR 0.77, 95% CI 0.71–0.84], a benefit which was noted in patients with and without a history of HF. These benefits were observed early (within 2 months) and therefore were unlikely attributable to modification of risk factors such as glycemia and body mass index (BMI). Moreover, there was a signal that SGTL2 inhibition may have renoprotective effects and indeed more pronounced benefits in patients with chronic kidney disease.^8,9^

This led to *CREDENCE* (canagliflozin—renal outcomes in diabetes and nephropathy) in 2019, a randomized placebo-controlled trial (n = 4401, mean age 63.0 years, follow-up 2.62 years) evaluating the effects of canagliflozin on renal outcomes among patients with T2DM and chronic kidney disease. Notably, this trial was the first to include patients with an estimate glomerular filtration rate (eGFR) 30–90 mls/min. The primary composite outcome of end-stage renal disease, doubling of serum creatinine, renal, or CV death was significantly reduced in the canagliflozin group (43.2 vs 61.2 per 1000 patient-years, *P* = 0.00001). Indeed, the benefits were so marked that the trial was terminated early on the recommendation of the data and safety monitoring committee.^10^

## Sodium-Glucose Cotransport 2 Inhibitors in Heart Failure

Taken in concert it became clear that SGLT2 inhibitors had a plethora of benefits beyond blood glucose control. Indeed, secondary analysis of these trials appeared to signal that the cardioprotective effects were independent of baseline HbA1c, suggesting a possible alternative mechanism of action of its cardioprotective effects.^8^

Following these landmark CV outcome trials, the first clinical trials into SGLT2i in HF were performed, the first of which was *DAPA-HF* (dapagliflozin—impact on patients with heart failure and reduced ejection fraction regardless of diabetes). This trial was unique in that it was the first to include nondiabetic patients and those with established HF, whereas previous CV outcome trials had only evaluated diabetic patients without HF. Importantly, the trial had a much wider inclusion criteria with respect to renal function, including patients with an eGFR as low as 30 mls/min.^11^

The primary composite outcome (worsening HF and CV death) was significantly lower in the treatment arm (16.3% vs 21.2%; *P* < 0.001; HR, 0.74; 95% CI, 0.65–0.85). Individually, each outcome was reduced; all-cause death (HR, 0.83; 95% CI, 0.71–0.97), CV death (HR, 0.82; 95% CI, 0.69–0.98), and HF hospitalization (HR, 0.70; 95% CI, 0.59–0.83). Moreover, dapagliflozin produced a significant improvement in quality of life outcome measures with a ≥5-point improvement in the Kansas City Cardiomyopathy Questionnaire symptom score (Odds ratio, 1.15; 95% CI, 1.08–1.23).

This trial was the first to demonstrate benefit in patients with HFrEF and, importantly, this benefit was seen in patients with and without T2DM.

The *EMPEROR-REDUCED* (empagliflozin outcome trial in patients with chronic heart failure and a reduced ejection fraction) trial subsequently demonstrated similar benefits with empagliflozin in patients with HF. This consolidated findings from DAPA-HF and suggested the effects weren’t specific to 1 drug type but rather a class effect of SGLT2 inhibition. Benefits were seen in patients with and without T2DM; however, compared *to DAPA-HF* this trial included arguably sicker patients, with a lower mean left ventricular ejection fraction (LVEF) of 27.7% (compared to 31.2% in *DAPA-HF*) and a lower exclusion threshold for renal function (20 mls/min/m^2^). Despite this, the trial still demonstrated a significant difference in the primary endpoint (composite of CV death or hospitalization for worsening HF) between empagliflozin and placebo (24.7% vs 19.4%, respectively; HR, 0.75; 95% CI, 0.65–0.86).^12^

Indeed, following the publication of these 2 landmark trials, SGLT2i’s rapidly became incorporated into clinical guidelines for the treatment of HFrEF internationally.^13^

Attention soon moved to evaluating the efficacy of SGLT2 inhibitors in HF with preserved ejection fraction (HFpEf). Historically these are a very difficult subset of HF patients to treat, with previously no effective pharmacotherapy proven in clinical trial data to reduce morbidity or mortality. Published in 2021, *EMPEROR-PRESERVED* (empagliflozin outcome trial in patients with chronic heart failure with preserved ejection fraction) enrolled 5988 patients (mean age 72 years, median follow-up 26.2 years) with symptomatic HF and LVEF >40% (mean LVEF was 54% with approximately a third of patients having an LVEF of 40–49%, a third having an LVEF of 50–59%). The primary outcome, a composite of CV death or HF hospitalization, was significantly reduced in the empagliflozin group (13.8% vs 17.1%; HR, 0.79; 95% CI, 0.69–0.90; *P* < 0.001). This was primarily driven by a 27% relative risk reduction in HF hospitalizations (8.6% vs 11.8% (HR, 0.73; 95% CI, 0.61–0.88). The treatment benefit was noted regardless of diabetes status. Moreover, benefit was maintained across the spectrum of LVEF in the study, except for patients with LVEF ≥60%, in whom an attenuated response was noted.^14^

Subsequently, the *DELIVER* (dapagliflozin evaluation to improve the lives of patients with preserved ejection fraction heart failure) trial evaluated the effect of dapagliflozin in patients with symptomatic HF with a LVEF >40%, with similarly positive reductions in the primary composite outcome of CV death or worsening HF (16.4% vs 19.5%; HR, 0.82; 95% CI, 0.73–0.92; *P* < 0.001).^15^

**FIGURE 1.**
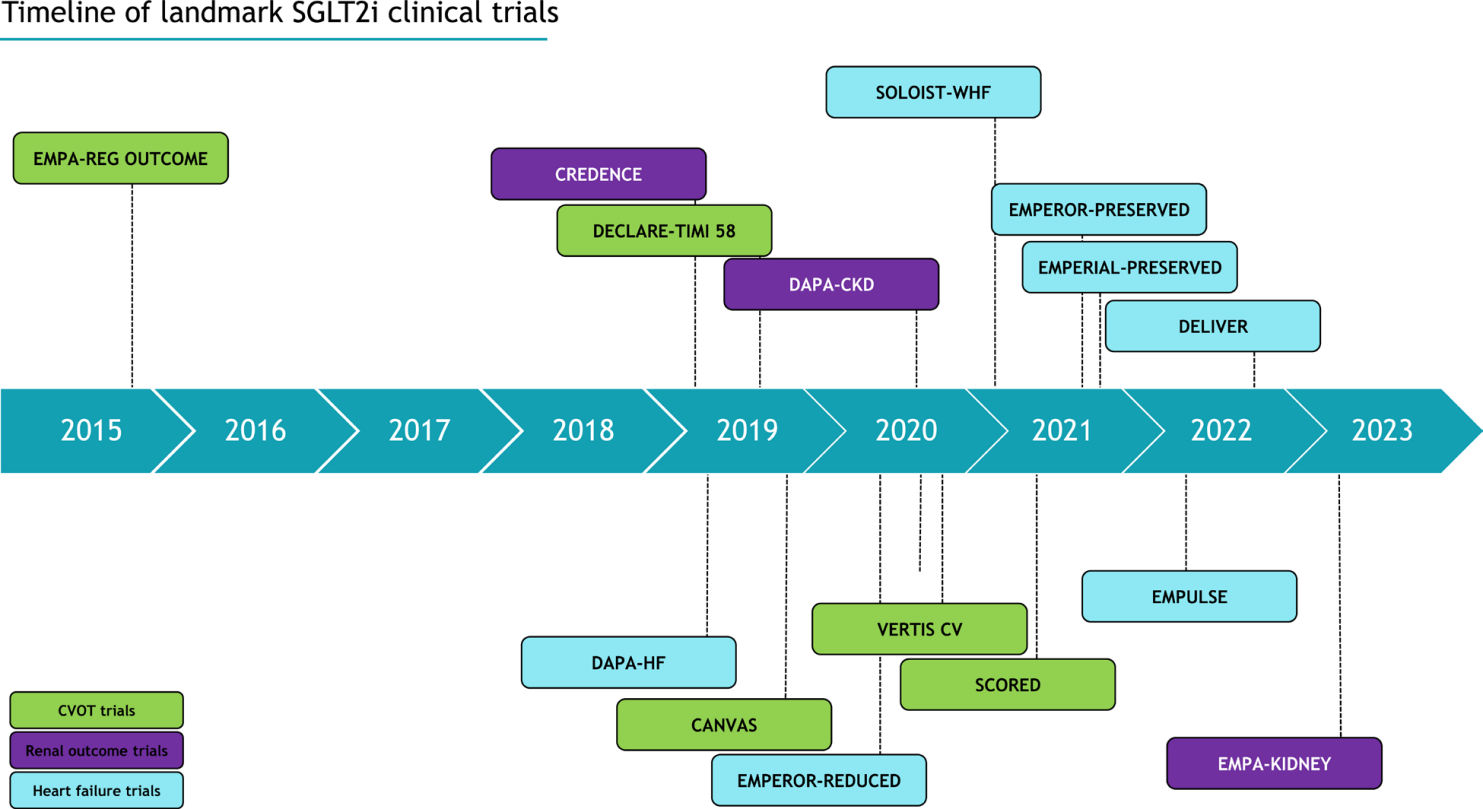
A summary timeline detailing landmark SGLT2 inhibitor clinical trials. Key CVOT trials include EMPA-REG OUTCOME (empagliflozin, cardiovascular outcomes, and mortality in type 2 diabetes), DECLARE-TIMI 58 (dapagliflozin and cardiovascular outcomes in type 2 diabetes), and VERTIS CV (cardiovascular outcomes with ertugliflozin in type 2 diabetes). Key renal outcome trials include CREDENCE (canagliflozin and renal events in diabetes with established nephropathy clinical evaluation), DAPA-CKD (dapagliflozin in patients with chronic kidney disease), SCORED (sotagliflozin in patients with diabetes and chronic kidney disease), and EMPA-KIDNEY (empagliflozin in patients with chronic kidney disease). Key heart failure trials include DAPA-HF (dapagliflozin in patients with heart failure and reduced ejection fraction), EMPEROR-REDUCED (cardiovascular and renal outcomes with empagliflozin in heart failure), EMPERIAL PRESERVED (effect of empagliflozin on exercise ability and symptoms in heart failure patients with reduced and preserved ejection fraction, with and without type 2 diabetes) and DEVILVER (dapagliflozin in heart failure with mildly reduced or preserved ejection fraction). Additionally, landmark trials in SGLT2 inhibition in acute heart failure include EMPULSE (The SGLT2 inhibitor empagliflozin in patients hospitalized for acute heart failure: a multinational randomized trial) and SOLOIST-WHF (sotagliflozin in patients with diabetes and recent worsening heart failure).

The culmination of this data^16–18^ has led to SGLT2 inhibitors’ rapid incorporation into international HF guidelines for the treatment of both HFrEF and HFpEF.^1^ A summary timeline of these trials is provided in Figure 1. Despite the robust evidence for its clinical efficacy, many questions remain unanswered regarding its mechanism of action, as its plethora of cardioprotective effects are not explained merely by the promotion of glycosuria.

This review seeks to explore, in detail, the possible mechanisms by which SGLT2 inhibition exerts direct and indirect cardiac effects.

## PHARMACOLOGY AND PHYSIOLOGY

**FIGURE 2.**
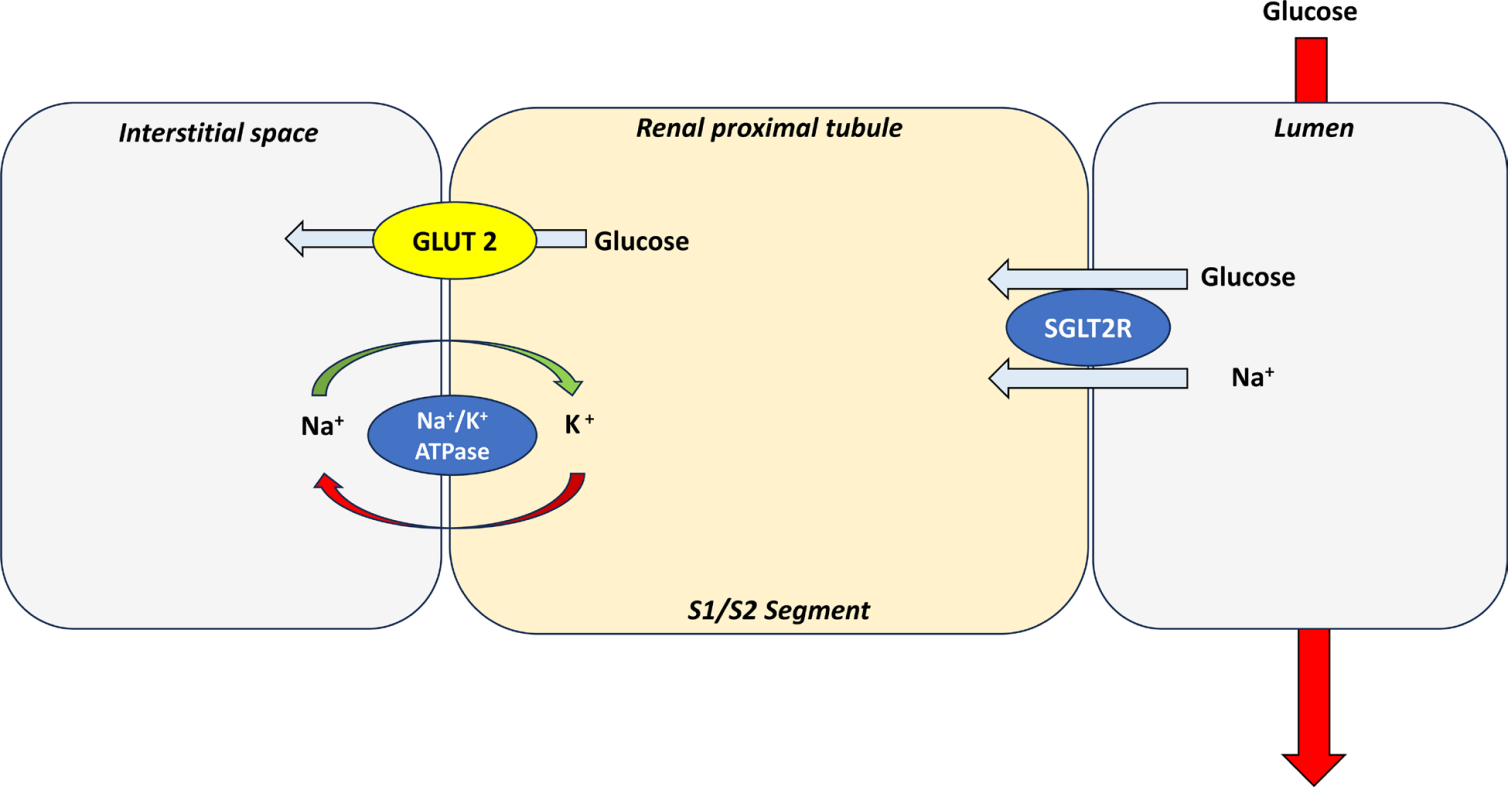
Drawing demonstrating the location and function of the SGLT2 receptor in the proximal convoluted tubule within the cortical nephron. Glucose is absorbed from the tubular lumen via SGLT2 receptor located on the luminal membrane surface with transport occurring against the concentration gradient. Glucose is further removed by passive diffusion via GLUT transporters with Na/K-ATPase cotransporters acting to maintain a sodium ion homeostasis.

The SGLT are a family of channel proteins that mediate the transport of glucose, osmolytes, ions, and amino acids. There are 2 receptors of interest, namely SGLT1 and SGLT2. SGLT1 receptors are found throughout the body, including the kidneys, intestine, heart, lungs, and skeletal muscle whereas SGLT2 receptors are confined to the kidney, pancreatic alpha cells, and the cerebellum. The SGLT2 receptor is a high affinity/high capacity protein with the ability to actively transport glucose and sodium at a 1:1 ratio against a concentration gradient and is responsible for 80–90% of the body’s glucose reabsorption compared to 10–20% facilitated by SGLT1 receptors^4^ (Fig. 2).

The antihyperglycaemic effect of SGLT2 inhibition is mediated by a reduction in the threshold for renal excretion of glucose. In a healthy kidney, this threshold for glycosuria is reached at 10 mmol/L (180 mg/dL) wherein inhibition of SGLT2 reduces this threshold reducing to 2.2 mmol/L (40 mg/dL). This promotes glycosuria, reduces serum glucose and improves insulin sensitivity. Furthermore, the antihyperglycaemic effect of SGLT2 inhibition is influenced by serum glucose as well as renal function, as its glycosuric effects are mediated by concentration and rate of glucose delivery to the proximal tubule in patients without diabetes, only a small amount of glycosuria and thus negligible lowering of circulating glucose levels are observed. Similarly, a reduction in eGFR results in an attenuation of the glucose-lowering effect due to a decreased rate of filtration and consequently, glycosuria. Indeed, this is demonstrated in studies where SGTL2 inhibition results in a 0.79% reduction in HbA1c, in eGFR ranges between 30–59 mls/min/1.73 m^2^, whereas this effect becomes negligible at a GFR <30 mls/min.^19^

Interestingly this contrasts with observed effects in HF outcomes in patients with HFrEF, where patients with a reduced GFR seemed to gain more benefit from the drug with respect to CV outcomes and HF hospitalizations.^20^

## CARDIAC MECHANISMS—DIRECT AND INDIRECT EFFECTS

An abundance of evidence has demonstrated the efficacy of SGLT2 inhibition in the treatment of HF across the spectrum of LVEF, independently of baseline diabetes status. Unlike its glycosuric effects, its efficacy in improving HF outcomes is not attenuated by declining renal function. Additionally, combined data from *DAPA-HF* and *EMPEROR-REDUCED* has demonstrated the clinical effects of the drug are noted relatively quickly, with an early separation in Kaplan–Meier curves for mortality noted in the early CV outcome trials. Therefore, it is unlikely that the effects observed are merely from classical risk factor modification of blood pressure, cholesterol, or HbA1c—which would typically take much longer to modify real-world risk.^21^

**FIGURE 3.**
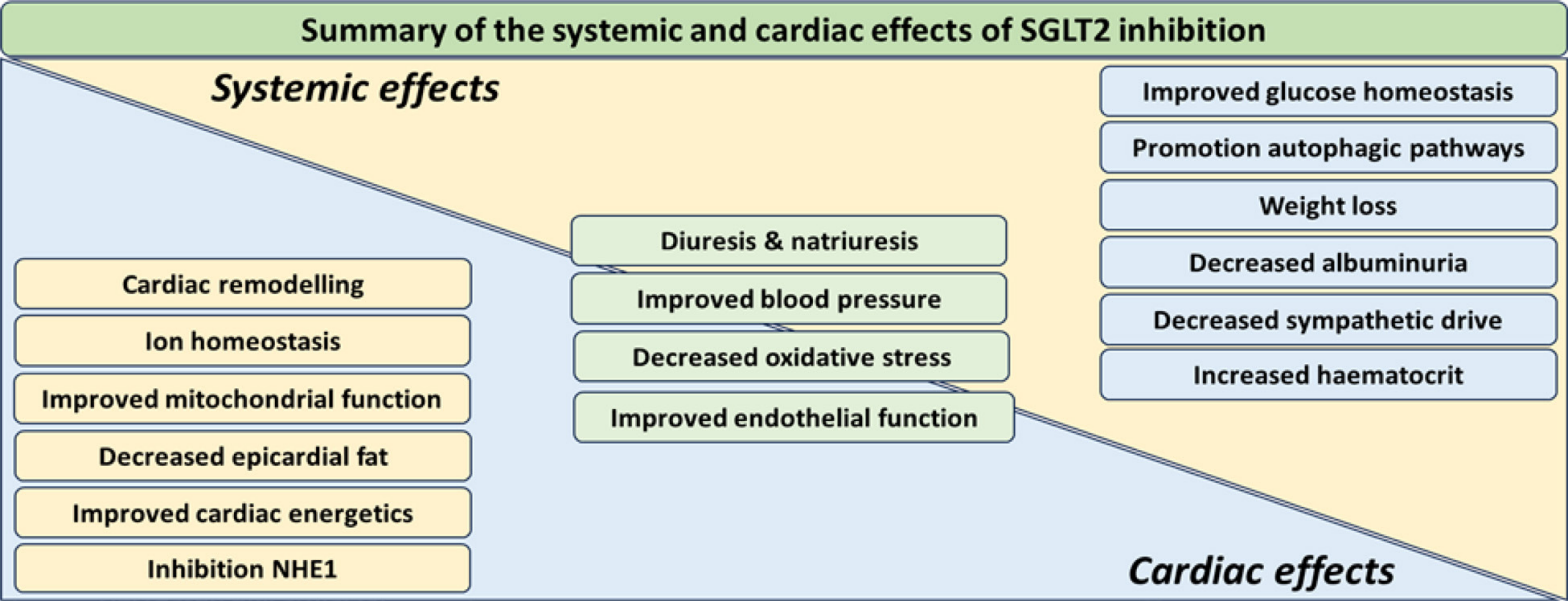
A summary of the systemic and cardiac effects of SGLT2 inhibition with overlapping synergistic effects demonstrated.

SGLT2 inhibitors confer a plethora of benefits not confined to the CV system alone (Fig. 3). Although many mechanistic questions remained unanswered, several theories have been postulated to explain its cardioprotective effects with both direct and indirect mechanisms proposed:

### Improved Cardiac Hemodynamics—Natriuretic Effects

SGLT2 inhibitors promote natriuresis secondary to glycosuria. The consequent osmotic effect may serve to reduce preload which, in a patient with HF, would serve to improve cardiac function. Indeed, secondary analysis of *EMPA-REG-OUTCOME* observed a degree of hemoconcentration which may be reflective of this volume contraction.^22^ Interestingly, these effects on erythrocyte mass and plasma cell volume are not noted when compared to standard diuretics such as hydrochlorothiazide.^23^

Further comparisons of mechanisms of action with respect to standard diuretics serve to identify some clues in differential mechanisms. Conventional diuretics differ from SGLT2 inhibitors in that they directly antagonize NaCI entry into the macula densa, whereas SGLT receptors are located in the proximal convoluted tubule. Therefore, their inhibition results in increased delivery of sodium to the macula densa which ultimately may not trigger the same degree of renin–angiotensin–aldosterone system and sympathetic stimulation.^4^

Furthermore, there is some evidence that SGLT2 inhibitors may preferentially promote interstitial fluid loss as opposed to plasma volume, not seen with conventional diuretics. Indeed, using mathematical modeling, Täger et al.^24^ demonstrated dapagliflozin produced a twofold greater reduction in interstitial fluid volume compared to blood volume, an effect not observed with bumetanide. Therefore, it may be that SGLT2 inhibition produces favorable cardiac effects, not seen in conventional diuretics, by differentially regulating interstitial fluid volumes and therefore not producing a deleterious exaggerated sympathetic response which is often seen with conventional diuresis in HF.

However, this may only be a part of the explanation and it is unlikely that SGLT2 inhibitors exert their pleotropic benefits merely by osmotic effects, particularly when similar cardioprotective benefits are not observed with standard diuretics.^24^ Indeed, it appears that their diuretic effects are short-lived and not corroborated by changes in other markers of volume regulation, such as NT-proBNP and albumin.^25,26^ Additionally, in large clinical trials, there was no observable reduction in maintenance diuretic dosing as a result of SGLT2 inhibition.^11^ Indeed, their osmotic effects have been demonstrated to be mainly mediated by glycosuria, not natriuresis with little change in short-term urinary sodium. Therefore, if their actions are merely due to a consequence of glycosuria, 1 would expect their effects to be potentiated in patients with diabetes and attenuated in renal dysfunction, neither of which are the case.

### Vascular Effects—Reduction of Arterial Stiffness

SGLT2 inhibition has been shown to produce reductions in blood pressure with reductions of 3–6 mmHg in systolic and 1–1.5 mmHg in diastolic noted in clinical trials.^17^ However, the modest improvements in systemic blood pressure do not explain the observed CV benefits. Indeed, if this were the case 1 would expect a reduction in atherothrombotic and stroke risk, not seen in clinical trial data. This has been reiterated in a meta-analysis.

Further data from post hoc analysis of clinical trial data has demonstrated reductions in pulse wave pressure following SGLT2 inhibition which is reflective of improved vascular function.^27^ Animal models have demonstrated that these changes in vascular resistance may be independent of systemic blood pressure changes a finding corroborated in an, albeit small, clinical study of patients with T2DM.^28^ Early preclinical data has suggested that possible mechanisms underpinning improved endothelial function may be mediated by activation of voltage-gated potassium channels, protein kinase G, increases in NO bioavailability and eNOS activation, as well as suppression of advanced glycation end products.^29^

### Improved Cardiac Energetics—Ketone Metabolism

It has been suggested that the failing heart up-regulates circulating ketones in an attempt to increase cardiac energy production. Traditionally, ketones are produced in the context of hypoglycemia as an alternative fuel source. Indeed, ketones have been shown to produce more energy per unit of oxygen compared to fatty acid or glucose which may have implications for energy preservation in the failing heart.^30,31^ It has been noted in patients with end-stage HF that ketone up-regulation is enhanced, interestingly administration of ketone body 3-hydroxybutyrate improves myocardial work without a consequent increase in myocardial energy demand. This has coined the “thrifty heart hypothesis” wherein the failing heart preferentially metabolizes ketones in order to improve efficiency of cardiac energetics.^32^ Interestingly in porcine models of ischemic nondiabetic HF, SGLT2 inhibition increased the utilization of branch-chain amino acids, fatty acids, and ketone bodies with concurrent improvement in left ventricular (LV) function and mitigation of HF remodeling.^33^ Conversely, in diabetic rat models of HF, SGLT2 inhibition reduced myocardial ketone body utilization despite increased levels of circulating ketone bodies with other models showing a blunting of decline in cardiac function independent of ketone utilization.^34^ Thus, it is unclear that the theory of “super-fueling” the heart is sufficient to explain the cardiac effects.

### Hemoconcentration and Erythropoiesis

In the original CV outcome trials, SGLT2 inhibition produced a noticeable increase in hematocrit and hemoglobin which initially was felt to be reflective of changes in volume status; however, accumulating evidence now suggests that this may be directly due to an increase in circulating erythropoietin (EPO) rather than hemoconcentration.^17,35^ Endogenous EPO is produced by the peritubular cells in the kidneys and is mediated by renal interstitial fibroblasts under the regulation of hypoxia-inducible factors. SGLT2 inhibition results in increased glucose delivery to the later parts of the proximal convoluted tubule which are partially absorbed in a compensatory fashion by SGLT1.^36^ This requires energy and produces a state of hypoxia which may induce the release of EPO as a compensatory measure. Theoretically, the increase oxygen delivery mediated by increased hemoglobin secondary to EPO production may help improve tissue delivery of oxygen and cardiac energetics in HF.

### Cardiovascular Risk Modification

SGLT2 inhibition has favorable effects with respect to modification of cardiac risk factors namely reduction in HbA1c, BMI, blood pressure, and serum uric acid.^35^ Hyperglycemia is a known risk factor for CV disease and its modification undoubtedly reduces cardiac risk. Although SGLT2i produces modest decreases in HbA1c, this effect is attenuated with renal dysfunction and normoglycemia. Indeed, recent evidence from DAPA-HF has demonstrated that the CV outcome benefits are independent of baseline diabetes.^17^ Despite the cardioprotective effects of improved glucose homeostasis it is unlikely that that this alone explains its cardiac effects.

Increased BMI is known to be an independent risk factor for atherosclerotic disease.^37^ Indeed, SGLT2 inhibitors have been shown to produce meaningful reductions in weight with small trials demonstrating reductions in visceral fat deposits reflecting that this is not merely a diuretic effect.^38,39^ Interestingly in large-scale trials, this reduction is modest and somewhat blunted over time with activation of compensatory mechanisms to maintain weight.^17^

Elevation of serum uric acid is associated with CV disease and adverse outcomes in HF. SGLT2 inhibition results in a modest decrease albeit in an eGFR-dependent fashion with its effects blunted below 60 mls/min. Again, this alone is likely insufficient to explain its cardiac effects and is likely a secondary, although favorable effect.^40^

SGLT2 inhibition has been shown to produce changes in blood pressure with reductions of 3–6 mmHg systolic and 1–1.5 mmHg diastolic noted in clinical trials which is comparable to low-dose hydrochlorothiazide.^41^ This is likely mediated through osmotic diuresis and mild natriuresis due to a higher tubular lumen glucose concentration and urine sodium excretion. Other mechanisms such as body weight reduction, suppression of sympathetic activation, improved endothelial function, and decreased arterial stiffness have all been suggested, indeed, all these mechanisms may be contributory.^19^

As discussed previously, this modest decrease in blood pressure does not explain the pronounced reduction in CV death and HF hospitalizations observed in clinical trials. Indeed, reduction in systemic blood pressure as a mechanism for reduced CV risk is unlikely, given it is understood that this modification in risk takes years and is usually reflected in reduced rates of stroke and atherothrombosis—neither of which are conferred by SGLT2 inhibition.^41,42^

Furthermore, the renoprotective effects noted from SGLT2i appear to outweigh the effects conferred from ACEi/ARBs, as intensive BP control (using ACEi/ARBs) in diabetic patients does not prevent end-stage renal failure. Therefore, suggesting that the modest BP changes produced by SGLT2i are not the mechanism by which it produces these favorable effects.^41,42,43^

### Anti-Inflammatory Effects

Wherein previously it was thought inflammation occurred secondary to HF, increasing evidence suggests that proinflammatory states may instead act as a driver of negative cardiac remodeling, promoting structural, and functional dysfunction.^44^

Oxidative stress and inflammation are known to cause endothelial dysfunction and promote adverse cardiac remodeling in models of MI. Increasingly, it is becoming apparent that SGLT2 inhibition exerts anti-inflammatory properties in both a direct and indirect fashion therefore mitigation of these pathways may explain their cardioprotective effects.^35^

Using both human myocardium and murine models of HFpEF, Kolijn et al.^45^ have demonstrated a significant reduction in markers of both oxidative stress (H_2_O_2_, GSH, and LPO) and inflammation (ICAM, VCAM, TNF-α, and IL-6) following treatment with SGLT2 inhibitors. Human clinical trials also reflect a strong signal of an anti-inflammatory effect with a recent meta-analysis showing a reduction in proinflammatory biomarkers (CRP, IL-6, and TNF-α) and oxidative stress (8-iso-prostaglandin F2 and 8-hydroxy-20-deoxyguanosine).^46^ Furthermore, real-world observational data has shown that SGLT2 inhibition produces noticeable changes in the redox status and antioxidant enzyme activity in the urine of T2DM patients.^47^

Multiple mechanisms have been postulated to explain SGLT2i anti-inflammatory effects with several theories highlighting shared systemic mechanisms.^19,40^ The secretion of adipokines from visceral adipose tissue is known to be associated with chronic inflammation and promotes metabolic dysfunction which in turn may contribute to cardiac dysfunction.^48^ In a small exploratory study by Garvey et al.^49^, patients with T2DM were treated with canagliflozin or glimepiride. At 52 weeks, compared to glimepiride, canagliflozin produced significant changes in circulating adipokines with a reduction in circulating leptin by 25% and increased adiponectin by 17%. Further corroboration of these findings can be found in recent studies utilizing cardiac magnetic resonance imaging which have shown that SGLT2 inhibition reduces epicardial adipose tissue which in turn may lead to reduced production of circulating adipokines.^50^

Similarly, uric acid is known to increase circulating levels of proinflammatory proteins via activation of the NLRP3 inflammasome which is a complex network of regulatory proteins which mediate the production of IL-1β and IL-18.^51^ Therefore, SGLT2 inhibition, which is shown to reduce serum uric acid levels, may indirectly promote anti-inflammatory pathways via its excretion.

SGLT2 inhibition may also exert direct effects on the NLRP3 inflammasome. In a rodent model of HF, Byrne et al.^52^ found that treatment with empagliflozin attenuated activation of the NLRP3 inflammasome. Similarly, Ye et al.^53^ found that treatment with dapagliflozin in diabetic mice, also suppressed NLRP3 levels. Interestingly, these effects have been borne out in clinical studies. In patients with diabetes treated with empagliflozin, Kim et al.^54^ noted a significant reduction in IL-1β, which, using ex vivo analysis, was found to be mediated by suppression of the NLRP3 inflammasome. Interestingly, they found that this was linked to up-regulation of ketone activity, perhaps suggesting an overlap in aforementioned mechanisms of action. Collectively, it remains unclear whether these are direct effects of SGLT2 receptor inhibition or if these are confined to the heart. Indeed, these actions may occur via-off target effects given that SGLT2 inhibition can affect inflammation and immune cells in extra-cardiac tissues. However, down-regulation of the NLRP3 inflammasome remains a plausible mechanism of action—both via direct and indirect inhibition.

### Myocardial Ion Homeostasis—Role of Na^+^/H^+^ Exchanger

Myocardial sodium homeostasis is central in maintaining cardiac contractility and the mitochondrial processes of oxidation–reduction, dysfunction in which is associated with the development of HF. Although SGLT2 receptors are minimally expressed in the heart, it has been implicated as having an important impact on myocardial ion homeostasis. The sodium-hydrogen exchanger (NHE) is a family of evolutionarily conserved antiporters consisting of ≥9 isoforms that are responsible for sodium/hydrogen exchange in the body. The NHE-1 is the predominant isoform in the heart where it regulates cardiomyocyte pH and volume and protects against ischemia-reperfusion injury.^55^

Inhibition of the cardiac sodium/hydrogen exchange (NHE-1) has been previously proposed as a mechanism by which SGLT2 inhibition produces direct cardiac effects. Indeed, in both in vitro mouse and human cardiomyocyte models, empagliflozin has been demonstrated to reduce intracellular Na via NHE-1 inhibition.^56,57^ There is some controversy with this theory as some studies have failed to replicate SGLT2-mediated NHE-1 inhibition in similar models. This may be explained by the tendency of normalizing pH to mitigate the inhibitory properties of SGLT2i on NHE-1, which is interesting, given that states of acidemia are associated with HF and there is some evidence that sicker patients clinically derive more benefit from SGLT2 inhibition.^17,58^ Indeed, further in vitro evidence towards this was presented by Peng et al.^59^ who demonstrated that pretreatment with ouabain (Na/K-ATPase pump inhibitor) was required for empagliflozin to exert favorable effects on cellular Na^+^/Ca^2+^ dysregulation, possibly by NHE inhibition. This suggests that the effects of SGLT2 inhibition may be dependent on ion homeostasis and pH. Although far from definitive, the fact that NHE-1 activity is increased in pathological conditions (eg, post-MI or HF), and its inhibition is cardioprotective, serves to highlight its possible role as a central mechanism in the cardioprotective effects of SGLT2 inhibition.

### Antifibrotic Effects

Myocardial fibrosis underpins negative cardiac remodeling seen in HFrEF and HFpEF, contributing to cardiac hypertrophy, cell necrosis, and inflammation. Increasing evidence is emerging showing SGLT2 inhibition may directly inhibit proinflammatory pathways responsible for cardiac fibrosis development and adverse cardiac remodeling in HF.^60,61^

In a rat model of MI, dapagliflozin was shown to have a suppressive effect on reactive oxygen species with reductions in myofibroblast and M2 macrophage infiltration. This has further been corroborated in rat models of diabetes and hypertension. These effects may be mediated by suppression of TGF-1 expression and Smad1, Smad2, Smad3 as well as type I and III collagen.^60,61^ Furthermore, this is likely due to direct effects on cardiac fibroblasts, given that the exposition of these cells to empagliflozin induces a more quiescent phenotype and a significantly attenuated cell-mediated extracellular matrix remodeling.^62^

These direct cardiac effects have been borne out in clinical studies evaluating the effect of SGLT2 inhibition on myocardial mass as evidenced on cardiac MRI. In *EMPA-HEART* (effect of empagliflozin on left ventricular mass in patients with type 2 diabetes mellitus and coronary artery disease), 97 patients with T2DM and coronary artery disease were randomized to treatment with placebo or empagliflozin. At 6 months, empagliflozin was noted to cause significant reductions in LV mass (2.6 vs 0.01 g/m^2^; 95% CI, −5.9 to −0.81g/m^2^; *P* = 0.01). Interestingly, in additional exploratory analyses, the changes in LV mass were not associated with blood pressure alterations suggesting a mechanistic effect independent of blood pressure changes.^63^

### Autophagy

Autophagy, a lysosome-mediated degradative pathway, has a key role in maintaining cardiac homeostasis and adaptation to stress and is noted to be impaired in both diabetes and HF. Autophagy-mediated clearance of damaged organelles reduces inflammasome activation and may in part explain the anti-inflammatory and antioxidant properties of SGLT2 inhibitors.^40^ Key proteins implicated in the activation of autophagic pathways include mammalian targets of rapamycin, sirtuins, and adenosine monophosphate-activated protein kinase. Several in vitro studies have since demonstrated the activation of these proteins in response to various SGLT2 inhibitors.^64,65^ Interestingly, these pathways may also have a dual effect in promoting anti-inflammatory pathways.

A recently published *EMPEROR* substudy conducted a proteomics analysis on a pooled sample from *EMPEROR-REDUCED* and *PRESERVED* (n = 1100). They identified significant changes in proteins associated with the regulation of autophagic flux in the heart, kidney, or endothelium, that is, IGFBP1, TfR1, EPO, follistatin, angiopoietin-related protein 4, and RBP2. Furthermore, they identified proteins involved in reduction of oxidative stress and improved myocardial mitochondrial health (TfR1, phospholipase A2, ANGLPTL4, IGFBP4, and uMtCK) as well as 4 proteins that promote cardiac repair and improved energetics (RBP2, IGFBP4, TGM2, and uMtCK).^66^ This data further supports the hypothesis that the cardioprotective effects of SGLT2 inhibitors are underpinned by their modulation of nutrient deprivation signaling and promotion of autophagy. Further work evaluating these pathways in the clinical setting as well correlating their changes to direct cardiac effects are needed.

## CONCLUSION

SGLT2 inhibitors have a demonstrable cardioprotective effect, unexplained by their ability to promote glycosuria and natriuresis. Several hypotheses have emerged over the years including modification of CV risk profile via weight reduction, improved glucose homeostasis, blood pressure control, and natriuretic effect; however, these mechanisms do not fully explain the potent effects of the drug demonstrated in large-scale randomized trials. Other mechanisms may be at play, specifically down-regulation of inflammatory pathways, improved myocardial sodium homeostasis, modulation of profibrotic pathways, and activation of nutrient deprivation signaling pathways promoting autophagic flux. Further work, both translational and experimental, is needed to further understand the complex mechanisms by which SGLT2 inhibitors exert their potent cardiac effects.

## ACKNOWLEDGMENTS

Patrick Savage is a clinical research fellow investigating the effects of SGLT2 inhibition in heart failure. His work is supported in the form of charitable funding received from the Belfast Heart Trust Fund. Chris Watson is supported by British Heart Foundation grant investigating novel therapeutics for diabetes and heart failure (PG/20/10424).
